# Patterns of trophic niche divergence between invasive and native fishes in wild communities are predictable from mesocosm studies

**DOI:** 10.1111/1365-2656.12360

**Published:** 2015-03-30

**Authors:** Thi Nhat Quyen Tran, Michelle C. Jackson, Danny Sheath, Hugo Verreycken, J. Robert Britton

**Affiliations:** ^1^ Department of Life and Environmental SciencesFaculty of Science and TechnologyBournemouth UniversityPooleBH12 5BBUK; ^2^ Department of Zoology and EntomologyCentre for Invasion BiologyUniversity of PretoriaGautengSouth Africa; ^3^ Research Institute for Nature and Forest (INBO)Kliniekstraat 25BrusselsB‐1070Belgium

**Keywords:** freshwater ecosystems, invasive species, stable isotope analysis, trophic niche width, trophic relationships

## Abstract

1. Ecological theory attempts to predict how impacts for native species arise from biological invasions. A fundamental question centres on the feeding interactions of invasive and native species: whether invasion will result in increased interspecific competition, which would result in negative consequences for the competing species, or trophic niche divergence, which would facilitate the invader's integration into the community and their coexistence with native species.
2. Here, the feeding interactions of a highly invasive fish, topmouth gudgeon *Pseudorasbora parva*, with three native and functionally similar fishes were studied to determine whether patterns of either niche overlap or divergence detected in mesocosm experiments were apparent between the species at larger spatial scales. Using stable isotope analysis, their feeding relationships were assessed initially in the mesocosms (1000 L) and then in small ponds (<400 m^2^) and large ponds (>600 m^2^).
3. In the mesocosms, a consistent pattern of trophic niche divergence was evident between the sympatric fishes, with niches shifting further apart in isotopic space than suggested in allopatry, revealing that sharing of food resources was limited. Sympatric *P. parva* also had a smaller niche than their allopatric populations.
4. In eight small ponds where *P*.* parva* had coexisted for several years with at least one of the fish species used in the mesocosms, strong patterns of niche differentiation were also apparent, with *P*.* parva* always at a lower trophic position than the other fishes, as also occurred in the mesocosms. Where these fishes were sympatric within more complex fish communities in the large ponds, similar patterns were also apparent, with strong evidence of trophic niche differentiation.
5. Aspects of the ecological impacts of *P*.* parva* invasion for native communities in larger ponds were consistent with those in the mesocosm experiments. Their invasion resulted in divergence in trophic niches, partly due to their reduced niche widths when in sympatry with other species, facilitating their coexistence in invaded ecosystems. Our study highlights the utility of controlled mesocosm studies for predicting the trophic relationships that can develop from introductions of non‐native species into more complex ecosystems and at larger spatial scales.

## Introduction

Biological invasions are often associated with native species declines which can modify biodiversity patterns and lead to biotic homogenization (Arim *et al*. 2006; Andreou *et al*. 2011). Predicting which introduced species will establish invasive populations and cause impacts remains a major ecological challenge (Barney & Whitlow 2008; Britton‐Simmons & Abbott 2008). With accelerating rates of biological invasions (Cohen & Carlton 1998; Jackson & Grey 2013) and horizon scanning exercises predicting current rates of introductions will continue (e.g. Gallardo & Aldridge 2015; Roy *et al*. 2014), there is an ongoing requirement for risk assessments to be based on empirical data (Copp *et al*. 2014). Dietary interactions with resident species frequently determine the outcome of introductions of non‐native species (Baiser, Russell & Lockwood 2010; Jackson *et al*. 2012), and they strongly influence the ecological impacts that develop in the native communities through, for example, predator–prey links (e.g. Woodford *et al*. 2005) and resource competition (e.g. Kakareko *et al*. 2013). Thus, comprehensive understandings of the trophic ecology of invasive species are essential for robust risk assessment (Britton, Davies & Brazier 2010; Britton, Gozlan & Copp 2011).

A long‐standing paradigm of freshwater invasion ecology is that ecological impacts often develop through increased interspecific competition for food resources occurring between invasive and sympatric native fishes (Gozlan *et al*. 2010a; Cucherousset *et al*. 2012). Adverse competitive effects from invasive fishes have been reported across a range of families covering different feeding guilds (e.g. Salmonidae, Crowl, Townsend & McIntosh 1992; Cyprinidae, Weber & Brown 2011; Cichlidae, Martin, Valentine & Valentine 2010). In contrast, in ecosystems where resources are not fully exploited, invasive species can occupy vacant dietary niches which facilitate their colonization by reducing competition with native populations (Shea & Chesson 2002; Jackson & Britton 2014). This niche partitioning is consistent with classical trophic niche theory which predicts that species occupy vacant niches and this enables their stable coexistence with other community members (Elton 1958; Chesson 2000; Kylafis & Loreau 2011). Moreover, the niche variation hypothesis predicts that under interspecific competition, populations become less generalized in their diet (Van Valen 1965), and thus, declines in niche width often occur in native populations following an invasion (Human & Gordon 1996; Thomson 2004; Olsson *et al*. 2009), with competing invasive species also expected to occupy smaller niches than their allopatric counterparts (Jackson *et al*. 2012). In contrast, increased competition for resources can result in larger trophic niches that enable species to maintain their energy requirements (Svanbäck & Bolnick 2007). These contrasting ecological theories on the consequences for the trophic niches of native species following an invasion can thus be tested using appropriate model species in order to better predict invasion outcomes and impacts.

Our aim was to test these contrasting ecological theories through using an invasive model fish species and three model native fish species. Objectives were to (i) quantify how invasion modified the trophic niche width and position of three model native fishes in experimental mesocosms (1000 L) using the species in allopatric and sympatric treatments; (ii) assess the trophic ecology of the invader and the three model native fishes across two other spatial scales: ‘small’ ponds (<400 m^−2^) of relatively low fish diversity and ‘large’ ponds (>600 m^−2^) of relatively high fish diversity; and (iii) test the hypothesis that the general patterns of trophic niche divergence or overlap detected between the invasive and native species at small spatial scales (mesocosms) are also evident at larger spatial scales and in systems of increased species diversity. The model invasive species was topmouth gudgeon *Pseudorasbora parva* (Temmnick & Schlegel), one of the 10 most invasive species in Europe that is of Southeast Asian origin (Britton & Gozlan 2013). Whilst a previous study suggested adverse ecological impacts for native fish occur through competitive interactions with *P*.* parva* (Britton, Davies & Harrod 2010), recent studies have suggested minimal sharing of food resources between *P*.* parva* and native species in many invaded fish communities (e.g. Jackson & Britton 2013, 2014). The model native fishes were common carp *Cyprinus carpio* L., tench *Tinca tinca* (L.) and three‐spined stickleback *Gasterosteus aculeatus* L. Note that as *C*.* carpio* has been present since at least the Middle Ages in the UK and in Belgium since the thirteenth century, and is considered naturalized in both (Verreycken *et al*. 2007; Britton *et al*. 2010), it was treated as a native species for the purposes of the study. In contrast, *P*.* parva* was introduced into Europe in only the 1960s (Gozlan *et al*. 2010b). All four fish species are omnivorous, with *P*.* parva, C*.* carpio* and *G*.* aculeatus* being bentho‐pelagic and *T*.* tinca* primarily being a benthic forager (www.fishbase.org).

## Materials and methods

### Mesocosm Experiment

The mesocosm experiments were completed in fibreglass ponds of *c*. 1000 L volume and 1 m depth that were situated in the open air on grass at a disused aquaculture site in southern England. The *P*.* parva*,* C*.* carpio*,* T*.* tinca* and *G*.* aculeatus* were each used in an allopatric treatment (eight individuals) and also in sympatric treatments of *P*.* parva* and *C*.* carpio*,* P*.* parva* and *T*.* tinca*, and *P*.* parva* and *G*.* aculeatus* (four individuals of each species). To prevent their reproduction, all *P*.* parva* and *G*.* aculeatus* were female and the *T*.* tinca* and *C*.* carpio* were all immature young‐of‐the‐year fish sourced from a local fish farm. Each treatment ran for 100 days between late July and October, a period providing sufficient time for isotopic turnover in the fish muscle (Jackson *et al*. 2014). The allopatric *P*.* parva*, allopatric *C*.* carpio* and sympatric *P*.* parva* and *C*.* carpio* treatments were run in this period in 2012 and used four replicates of each. A further allopatric *P*.* parva* treatment plus allopatric *T*.* tinca* and *G*.* aculeatus*, and sympatric *P*.* parva* and *T*.* tinca* and *G*.* aculeatus* treatments were run in 2013 and were replicated three times. All mesocosms were established 1 month prior to the fish being introduced by filling them with water from a nearby fishless pond. Each was provided with a gravel (*c*. 6 mm diameter) substrata (1·5 cm depth), provided with fish refuge structures (two open‐ended circular plastic tubes of 15 cm length and 6 cm diameter) and a native pond lily (*Nymphoides peltata*; uniform wet mass was 10 ± 1 g), and was seeded with Chironomidae, *Asellus aquaticus* and *Gammarus pulex* (20 of each) to enable establishment of a macro‐invertebrate community. Each mesocosm was covered with 20‐mm nylon mesh to prevent access for predators. During the 100‐day period in both years, water temperatures were recorded between 7·6 and 19·2  °C (mean ± SE: 13·6 ± 0·9  °C; measured hourly using a data logger).

Following the 100th day after the fish were introduced, each mesocosm was partially emptied of its water using buckets and the fish recaptured using hand nets. They were taken to the laboratory where they were measured (fork length, *L*_F_, nearest mm) and a sample of dorsal muscle taken for stable isotope analysis. Putative fish food resources (algae, benthic invertebrates and zooplankton; *n *=* *3–9 of each) were also taken from each mesocosm for this purpose. All samples were oven‐dried to constant weight at 60  °C before analysis at the Cornell Isotope Laboratory, New York, USA. The initial data outputs were in the format of delta (δ) isotope ratios expressed per mille (‰). To enable combining data from the replicates in each treatment for subsequent analyses and comparisons between treatments, δ^13^C and δ^15^N were corrected due to some significant differences in the resource isotopic data between mesocosms and some treatments being run in 2012 and others in 2013. For δ^15^N, correction was made by calculating trophic position (TP) using TP_i_ = [(δ^15^N_i_ − δ^15^N_base_)/3·4] + 2, where TP_i_ is the trophic position of the individual fish, δ ^15^N_i_ is the isotopic ratio of that fish, δ ^15^N_base_ is the isotopic ratio of the primary consumers (i.e. the ‘baseline’ invertebrates), 3·4 is the fractionation between trophic levels, and 2 is the trophic position of the baseline organism (Post 2002). For δ^13^C, correction was according to the following: δ^13^Ccorr = δ^13 ^C_i_ − δ^13^C_meaninv_/CR_inv_, where δ^13^C_corr_ is the corrected carbon isotope ratio of the individual fish, δ^13 ^C_i_ is the uncorrected isotope ratio of that fish, δ^13^C_meaninv_ is the mean invertebrate isotope ratio (the ‘baseline’ invertebrates), and CR_inv_ is the invertebrate carbon range (δ^13^Cmax–δ^13^Cmin; Olsson *et al*. 2009).

The corrected stable isotope data were initially used in linear mixed models to test differences in trophic position between sympatric species and identify how allopatry and sympatry affected the trophic position of each species. Prior to model construction, assumptions of normality of residuals and homoscedasticity were checked, and response variables were log‐transformed as necessary. Species were entered separately into models according to their treatments so, for example, *P*.* parva* were present in models as allopatric *P*.* parva*, and in sympatry with *C*.* carpio*,* T*.* tinca* and *G*.* aculeatus*. Due to the format of these data (i.e. treatments with replicates, with replicates providing data from individual fish), using data from individual fish as true replicates would inflate the residual degrees of freedom; thus, models were fitted with mesocosm as a random effect (Dossena *et al*. 2012). The significance of each treatment on the trophic position of each species was assessed by starting with the most complex model and then simplifying by removing non‐significant terms using maximum‐likelihood ratio tests due to different fixed effects structures (i.e. different degrees of freedom). Final models were refitted using restricted maximum likelihood to determine the parameter estimates, with differences in trophic positions by species and treatment in the final model determined using estimated marginal means and multiple comparison post hoc analyses (general linear hypothesis test). This linear mixed model approach was also used to test for differences in fish length between sympatric species and to identify length differences for each species between allopatry and sympatry.

The corrected stable isotope data were then used to calculate the standard ellipse area (SEA) as measure of niche width for each species in each treatment using the SIAR package (Jackson *et al*. 2011) in the R computing program (R Core Team 2013). SEA is a bivariate measure of the distribution of individuals in trophic space; each ellipse encloses *c*. 40% of the data and, therefore, represents the core dietary niche, indicating typical resource use within a species or population (Jackson *et al*. 2011, 2012). To account for variation in sample sizes, we calculated a Bayesian estimate of SEA (SEA_B_) using Markov chain Monte Carlo simulation with 10^4^ iterations for each group (Jackson *et al*. 2011; R Core Team 2014). Should there be a situation where the SEA_B_ overlapped between the sympatric fishes within a treatment, then the area and extent of overlap (%) were also calculated to indicate the extent of resource sharing.

### Small Ponds

Eight small ponds, adjacent to the mesocosm ponds, were sampled in August 2013. These ponds were 30–40 m in length, 8–10 m in width and had a maximum depth of 2 m. Extensive beds of the submerged macrophyte *Elodea canadensis* were present in each. Although previously used for fish culture, none of the ponds had been used for this purpose since the mid‐2000s and they had not been sampled or manipulated since then, and the composition of their fish communities varied. In four ponds, only *P*.* parva* and *G*.* aculeatus* were present (a–d), in two ponds, *P*.* parva* and *T*.* tinca* were present (e and f), and in the final two ponds, *P*.* parva, T*.* tinca* and *G*.* aculeatus* were present (g and h). Fish samples were collected using rectangular fish traps comprising of a circular alloy frame of length 107 cm, width and height 27·5 cm, mesh diameter 2 mm and with funnel‐shaped holes of 6·5 cm diameter at either end to allow fish entry and hence their capture. Each trap was baited with five fishmeal pellets of 21 mm diameter (Jackson *et al*. 2014) and were fished in triplicate in each pond and set in the morning (*c*. 9 am) and lifted *c*. 2 h later. Following lifting of the fish traps, all fish were removed and identified to species level. Random subsamples (minimum *n* = 8) of each species were taken back to the laboratory where they were euthanized using an overdose of anaesthetic (MS‐222), measured (*G*.* aculeatus*: total length; other fish species: fork length; all nearest mm) and a sample of dorsal muscle taken. At the same time as the fish sampling, sweep nets were used to capture macro‐invertebrates. Samples for stable isotope analysis were prepared and analysed as per the mesocosms.

The trophic position of each individual fish was then calculated for each fish per pond (using the trophic position equation outlined in the mesocosm subsection). These data then tested between species in linear mixed effects models as per the mesocosms, with models constructed for ponds a to d, e and f, and g and h. In the models, ‘pond’ was fitted as the random variable and fish length was included as a covariate in the final model if its effect on trophic position was significant. The significance of differences in trophic positions between species was determined by pairwise comparisons of estimated marginal means, adjusted for multiple comparisons by Bonferroni corrections. We then calculated SEA_B_ for each population in each individual pond, and the trophic overlap between the sympatric species (where it occurred), using the methodology already described for mesocosms.

### Large Ponds

The three wild fish communities used in the study that were invaded by *P*.* parva* were sampled in March 2013. Two were in Belgium (Pond a: 51 °2′17·64″ N 4 °10′54·86″ E, 600 m^2^; Pond b: 50 °2′59′3·35″ N 5 °20′10·52″ E; 1900 m^2^) and the other in Wales (Pond c: 51 °41′10·0″ N 4 °12′06·00″ W; 3000 m^−2^). In these ponds, the consistent similarity with the mesocosms and the small ponds was that in Pond a, *P*.* parva* coexisted with *G*.* aculeatus;* in Pond b, they coexisted with *G*.* aculeatus* and *C*.* carpio;* and in Pond c, they coexisted with *T*.* tinca*. Fish sampling incorporated electric fishing, seine nets, fish traps and fyke nets, with macro‐invertebrate samples collected using sweep nets. Following their capture, the fish were euthanized and returned to the laboratory where data collection and dorsal muscle samples were taken as per the mesocosms and small ponds. Baseline differences were not examined between ponds as the aim was to compare niches within, rather than among, each community. As the water temperatures were still <8  °C at the time of sampling, the dorsal muscle samples of the fish reflected their values at the end of their 2012 growth season (Perga & Gerdeaux 2005).

The fish and macro‐invertebrate samples were then prepared and analysed for their stable isotopes of δ^13^C and δ^15^N as already described for the mesocosms and small ponds. The trophic position of each individual (using the TP equation outlined of correcting mesocosm data) was then determined, with these data then tested between species in generalized linear models (GLM), as the data were not normally distributed; the independent variable was trophic position and the covariate was fish length due to significant differences in lengths between some species (cf. Results). The significances of the differences in trophic position between species were determined by pairwise comparisons of estimated marginal means, adjusted for multiple comparisons by Bonferroni corrections. Calculations of SEA_B_ and the extent of niche overlap (%) were then completed as already described for the mesocosms.

## Results

### Mesocosms

The starting length ranges of the fish used across the experiment varied (Table 1), with the effect of species on length being significant (*P *<* *0·01). Pairwise comparisons revealed there was no significant difference in the starting lengths of the sympatric *P*.* parva* and *G*.* aculeatus* (mean difference 0·47 mm, *P *>* *0·05) or between their allopatric vs. sympatric treatments (*P*.* parva*: mean differences 0·54–0·71 mm, *P *>* *0·05; *G*.* aculeatus* mean difference 3·6 mm, *P *>* *0·05). Length differences between sympatric *P*.* parva* and *C*.* carpio* were also not significant (0·71 mm, *P *>* *0·05), but were between sympatric *P*.* parva and T*.* tinca* (22·0 mm, *P *<* *0·01). There were no significant differences in lengths between the allopatric and sympatric treatments of *C*.* carpio* (0·48 mm, *P *>* *0·05) and *T. tinca* (0·88 mm, *P *>* *0·05). Of these original fish, 92% were recovered at the conclusion of the 100 days, including all *C*.* carpio* and *T*.* tinca*, with lower recovery rates for *G*.* aculeatus* and some *P*.* parva* treatments (Table 1).

**Table 1:** Numbers, lengths and stable isotope data and metrics of the fish recovered from the mesocosm experiments

Context	*n*	Mean length (mm)	Mean δ^13^C (‰)	Mean TP	SEA_B_ (‰^2^)
Allopatric *P*.* parva*	43	32·3 ± 1·2	−27·60 ± 0·16	3·56 ± 0·04	1·01 (0·90–1·10)
Allopatric *G*.* aculeatus*	18	31·8 ± 1·4	−28·71 ± 0·39	4·04 ± 0·05	1·32 (1·09–1·49)
Allopatric *T*.* tinca*	24	69·5 ± 1·1	−24·04 ± 0·14	4·60 ± 0·07	0·77 (0·66–0·87)
Allopatric *C*.* carpio*	24	54·2 ± 1·1	−20·40 ± 0·12	4·39 ± 0·02	1·06 (0·91–1·18)
Sympatric *G*.* aculeatus*	8	35·4 ± 1·2	−28·56 ± 0·45	3·46 ± 0·03	1·69 (1·26–1·99)
Sympatric *P*.* parva*	11	34·9 ± 2·8	−26·76 ± 0·29	3·85 ± 0·11	0·79 (0·62–0·92)
Sympatric *T*.* tinca*	12	70·4 ± 1·4	−23·82 ± 0·25	4·77 ± 0·06	1·36 (1·08–1·56)
Sympatric *P*.* parva*	12	48·4 ± 1·1	−27·93 ± 0·28	3·17 ± 0·03	0·75 (0·59–0·86)
Sympatric *C*.* carpio*	16	56·1 ± 1·4	−21·32 ± 0·31	4·50 ± 0·06	0·88 (0·72–1·01)
Sympatric *P*.* parva*	16	49·3 ± 1·2	−27·01 ± 0·26	3·38 ± 0·07	0·96 (0·78–1·09)

SEA_c_, standard ellipse area; TP, trophic position.

Error around the mean is standard error; values in parentheses are confidence intervals.

The final trophic positions of the fish species also varied across the experiment (Table 1). Length had no significant effect on trophic position (*P *=* *0·69) and so was removed from the final model (Table 2). There were significant differences in trophic position between sympatric species (*P *<* *0·01), with *P*.* parva* always at the lower trophic position, but not between each species in allopatry and sympatry (Table 2).

**Table 2:** Outputs of the final linear mixed model testing the differences in trophic position between the species across the mesocosm experiment, where mesocosm was the random effect on the intercept

Final model: trophic position ~ species × experimental treatment (AIC = 51·71; log‐likelihood = 47·71; *P *<* *0·01)		
Pairwise comparison	Mean difference (estimated marginal means)	
Allopatric *P*.* parva*	*P*.* parva* sympatric with *C*.* carpio*	0·08, *P *>* *0·05
	*P*.* parva* sympatric with *T*.* tinca*	0·18, *P *>* *0·05
	*P*.* parva* sympatric with *G*.* aculeatus*	0·29, *P *>* *0·05
Allopatric *C*.* carpio*	*C*.* carpio* sympatric with *P*.* parva*	0·11, *P *>* *0·05
Allopatric *T*.* tinca*	*T*.* tinca* sympatric with *P*.* parva*	0·18, *P *>* *0·05
Allopatric *G*.* aculeatus*	*G*.* aculeatus* sympatric with *P*.* parva*	0·08, *P* > 0·05
*P*.* parva* in sympatry with *C*.* carpio*		1·11, *P *<* *0·01
*P*.* parva* in sympatry with *G*.* aculeatus*		0·76, *P *<* *0·01
*P*.* parva* in sympatry with *T*.* tinca*		1·60, *P *<* *0·01

**Figure 1:**
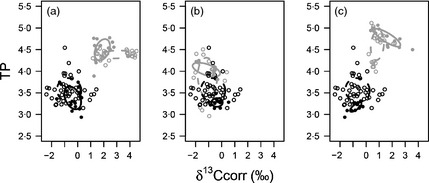
Grey ellipses enclose the trophic niche of *Cyprinus carpio* (a), *Gasterosteus aculeatus* (b) and *Tinca tinca* (c) in the experimental mesocosms in the presence (solid grey) and absence (dashed grey) of *Pseudorasbora parva*. Black ellipses enclose the trophic niche of sympatric (solid black) and allopatric (dashed black) populations of *P*.* parva*. Note there is no overlap of the sympatric invasive *P*.* parva* (solid black) and native fish (solid grey) trophic niches in any of the treatments.

Standard ellipse areas (SEA_B_) revealed that in sympatry, *C*.* carpio* and *T*.* tinca* had larger trophic niches than *P*.* parva* (Table 1) with their trophic niches showing no overlap (Fig. 1). In contrast, *P*.* parva* had a slightly larger trophic niche than *G*.* aculeatus* (Table 1) although there was also no overlap in their trophic niches (Table 1; Fig. 1). The trophic niche size of *P*.* parva* was always smaller in sympatry than allopatry (Table 1). Whilst the native fishes had larger niches in sympatry, these changes were not significant as their errors overlapped (Table 1). Whilst the allopatric niches of *P*.* parva* and *G*.* aculeatus* overlapped, in sympatry, this was not evident due to niche shifts in both species (Fig. 1b).

### Small Ponds

**Figure 2:**
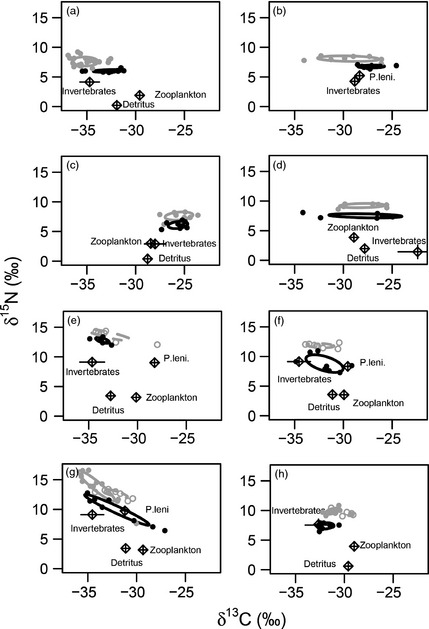
Food web structure in the eight small ponds, where the only fish present were *Pseudorasbora parva* (all ponds), *Gasterosteus aculeatus* (ponds a, b, c, d, g, h) and *Tinca tinca* (ponds e, f, g, h). Ellipses enclose the trophic niche of *P*.* parva* (solid black), *G*.* aculeatus* (solid grey) and *T*.* tinca* (dashed grey). Circular data points represent individual *P*.* parva* (closed black), *G*.* aculeatus* (closed grey) and *T*.* tinca* (open grey). Mean values (± standard error, *n* = 1–8) of resources are represented by open diamonds. Invertebrates included mayflies, *Gammarus pulex, Asellus aquaticus* and Chironomids. P.leni. = signal crayfish, *Pacifastacus leniusculus*. Note there is no overlap between invasive *P*.* parva* (black) and native fish (grey) trophic niches in any pond.

In the small ponds, the effect of fish species on trophic position was significant in all linear mixed models, but fish length was not and so was removed from final models. In ponds a–d, where only *P*.* parva* and *G*.* aculeatus* were present, *G*.* aculeatus* occupied a significantly higher trophic position than *P*.* parva* (mean difference 0·45 ± 0·04; *P *<* *0·01; Table 3). They also had a larger trophic niche in three of the four ponds (Table 3), with no overlap in their niche with *P*.* parva* (Fig. 2a–d). In ponds e and f, with only *P*.* parva* and *T*.* tinca* present, *T*.* tinca* occupied a significantly higher trophic position than *P*.* parva* (mean difference 0·58 ± 0·10; *P *<* *0·01; Table 3). Their trophic niche sizes varied between the two ponds (Table 3), with negligible trophic overlap (Fig. 2e,f). In ponds g and h, where *P*.* parva, T*.* tinca* and *G*.* aculeatus* were present, the trophic position of *P*.* parva* was significantly lower than *G*.* aculeatus* and *T*.* tinca* (*P*.* parva*/*G*.* aculeatus*: mean difference: 0·99 ± 0·16, *P *<* *0·01; *P*.* parva*/*T*.* tinca*: mean difference 0·73 ± 0·18, *P *<* *0·01); Table 3). There was also no overlap in the trophic niches of these fishes (Table 4; Fig. 2g,h).

**Table 3:** Sample sizes, fish lengths and stable isotope metrics of *Pseudorasbora parva* and the native fishes across the small ponds, where TP = trophic position, SEA_B_ is an estimate of standard ellipse area (trophic niche size), error around the mean is standard error, and values in parentheses are the confidence intervals of SEA_B_

Pond	Species	*n*	Mean δ^13^C	Mean TP	SEA_B_ (‰^2^)
a	*G*.* aculeatus*	22	−35·19 ± 0·28	3·26 ± 0·05	3·26 (2·75–3·66)
	*P*.* parva*	10	−32·78 ± 0·46	2·80 ± 0·02	2·32 (2·17–2·70)
b	*G*.* aculeatus*	8	−29·50 ± 1·03	3·45 ± 0·05	5·68 (5·22–6·67)
	*P*.* parva*	8	−27·14 ± 0·44	3·07 ± 0·02	2·27 (2·10–2·65)
c	*G*.* aculeatus*	8	−25·56 ± 0·47	3·39 ± 0·07	3·38 (2·49–3·97)
	*P*.* parva*	8	−25·67 ± 0·33	2·95 ± 0·06	2·67 (1·76–2·77)
d	*G*.* aculeatus*	8	−28·08 ± 0·76	3·76 ± 0·03	3·92 (2·88–4·58
	*P*.* parva*	8	−27·74 ± 1·22	3·26 ± 0·03	6·00 (4·44–7·01)
e	*T*.* tinca*	8	−32·89 ± 0·74	3·41 ± 0·10	4·35 (3·21–5·07)
	*P*.* parva*	8	−33·60 ± 0·23	3·15 ± 0·05	1·72 (12·8–2·02)
f	*T*.* tinca*	8	−32·35 ± 0·45	3·96 ± 0·04	2·54 (1·87–2·97)
	*P*.* parva*	8	−31·93 ± 0·63	3·06 ± 0·14	6·68 (4·95–7·84)
g	*G*.* aculeatus*	13	−34·12 ± 0·50	3·88 ± 0·20	6·11 (4·89–7·00)
	*T*.* tinca*	8	−32·32 ± 0·45	3·52 ± 0·07	2·84 (2·10–3·31)
	*P*.* parva*	8	−32·02 ± 1·08	2·70 ± 0·25	7·89 (5·82–9·25)
h	*G*.* aculeatus*	8	−31·25 ± 0·23	3·13 ± 0·05	1·81 (1·34–2·12)
	*T*.* tinca*	6	−30·10 ± 0·36	3·06 ± 0·06	2·30 (1·61–2·70)
	*P*.* parva*	8	−31·90 ± 0·27	2·40 ± 0·05	1·99 (1·48–2·32)

**Table 4:** Sample sizes, fish lengths and stable isotope metrics of *Pseudorasbora parva* and the native fishes across the wild ponds

Pond	Other fishes present	Species	*n*	Mean length (mm)	Mean δ^13^C (‰)	Mean TP	SEA_B_ (‰^2^)
a	*Carassius gibelio* a *; Rhodeus amarus* a *; Scardinius erythropthalmus; Pungitius pungitius*	*P*. *parva*	10	73·6 ± 2·2	−38·58 ± 0·13	3·56 ± 0·06	1·68 (1·29–1·95)
		*G*.* aculeatus*	9	42·9 ± 1·5	−39·64 ± 0·23	3·39 ± 0·08	2·70 (2·04–3·12)
b	*Carassius gibelio* a *; Rhodeus amarus* a *; Scardinius erythropthalmus; Blicca bjoerkna; Leucaspius delineates* a *; Rutilus rutilus*	*P*.* parva*	10	72·1 ± 2·3	−35·84 ± 0·41	4·08 ± 0·03	1·23 (0·94–1·43)
		*G*.* aculeatus*	10	48·4 ± 1·0	−38·84 ± 0·31	3·27 ± 0·12	3·89 (2·99–4·52)
		*C*.* carpio*	10	70·3 ± 2·7	−34·99 ± 0·35	3·88 ± 0·10	4·05 (3·12–4·70)
c	*Carassius auratus* a *; Scardinius erythropthalmus*	*P*.* parva*	20	63·0 ± 3·9	−25·51 ± 0·12	3·18 ± 0·02	0·92 (0·77–1·03)
		*T*.* tinca*	12	95·7 ± 10·1	−27·01 ± 0·57	3·52 ± 0·05	3·49 (3·32–4·01)

Error around the mean is standard error.

a These ‘other’ species are non‐native to the country.

### Large Ponds

**Figure 3:**
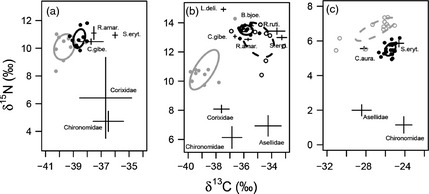
Food web structure in the three natural large pond communities in Belgium (a and b) and Wales (c). Ellipses enclose the trophic niche of *Pseudorasbora parva* (solid black), *T*.* tinca* (dashed grey), *Cyprinus carpio* (dashed black) and *Gasterosteus aculeatus* (solid grey). Data points represent individual *P*.* parva* (closed black), *T*.* tinca* (open grey), *C*.* carpio* (open black) and *G*.* aculeatus* (closed grey). Other community members are represented by means ± standard error (*n* = 3–14). C.aura. = goldfish, *Carassius auratus*; S.eryt. = common rudd, *Scardinius erythropthalmus*; C.gibe. = gibel carp, *Carassius auratus*; L.deli. = sunbleack, *Leucaspius delineates*; R.ruti. = roach, *Rutilus rutilus*; B.bjoe. = silver bream, *Blicca bjoerkna*; R.amar. = European bitterling, *Rhodeus amarus*. Note there is minimal niche overlap of invasive *P*.* parva* (solid black) and the model native fish, except *C*.* carpio* (dashed black), across all three ponds.

In the natural pond communities, the samples from the fish communities compromised of different numbers of species of varying lengths (Table 4). In Pond a, whilst *P*.* parva* were significantly larger than *G*.* aculeatus* (*F*_1,17_ = 8·69, *P *<* *0·01), *G*.* aculeatus* had the larger trophic niche, with only a small degree of overlap between the two species (5% and 2% of *P*.* parva* and *G*.* aculeatus* niches were shared respectively; Fig. 3a). There was no significant difference in the trophic position of the two species when length was taken into account (Wald χ^2^ = 1·03, *P *>* *0·05; Table 4a). In Pond b, *P*.* parva* were again significantly larger than *G*.* aculeatus* (*F*_1,18_ = 9·01, *P *<* *0·01), with a smaller trophic niche (Table 4) that did not overlap at all with *G*.* aculeatus* (Fig. 3b). The trophic position of *P*.* parva* was significantly higher than *G*.* aculeatus* (Wald χ^2^ = 19·02, *P *<* *0·01) (*P *<* *0·05; Table 4b). In this pond, *P*.* parva* and *C*.* carpio* were not significantly different in length (*F*_1,18_ = 1·94, *P *>* *0·05), with *C*.* carpio* having a larger trophic niche that overlapped strongly with *P*.* parva* (84 and 11% of *P*.* parva* and *C*.* carpio* niches were shared, respectively; Table 4b; Fig 3b). In Pond c, *T*.* tinca* were significantly larger than *P*.* parva* (*F*_1,30_ = 20·35, *P *<* *0·01) and had a larger, distinct trophic niche (Table 4, Fig. 3c). *Tinca tinca* had a significantly higher mean trophic position than *P. parva* (Wald χ^2^ = 10·86, *P *<* *0·01) (Table 4; Fig. 3c).

## Discussion

*Pseudorasbora parva* is a highly invasive species in Europe, capable of establishing invasive populations from small numbers of introduced individuals (Britton & Gozlan 2013). Here, patterns of trophic niche divergence detected between *P*.* parva* and three coexisting native fishes in mesocosms were generally consistent with patterns observed in natural ponds where the fish communities comprised of up to nine species. This finding supports our hypothesis that where general patterns of trophic niche divergence are detected between the invasive and native species at small spatial scales, these will also be evident at larger spatial scales and in systems of increased species diversity.

The trophic niches of the allopatric fish populations in the mesocosms indicated the potential for the diet of *P*.* parva* and *G*.* aculeatus* to overlap substantially. However, due to niche shifts of both species when in sympatry, niche divergence was apparent instead. Similarly, the niche of sympatric populations of *P*.* parva* and *T*.* tinca* shifted apart and so became more divergent in sympatry. These outputs are consistent with Jackson & Britton (2014) who found similar patterns of trophic niche divergence between *P*.* parva*,* C*.* carpio* and *Pacifastacus leniusculus* across six established small ponds in southern England. They are, however, contrary to Declerck *et al*. (2002), who found that there were no significant differences in native fish and invasive *P*.* parva* diets in two ponds according to stomach contents analyses, with strong probabilities of interspecific competition occurring. Our outputs were also contrary to Britton, Davies & Harrod (2010) who used stable isotope analysis to reveal resource sharing between *P*.* parva*, roach *Rutilus rutilus* and *C*.* carpio* in an invaded fishing lake in England. In the latter study, *P*.* parva* population abundance was extremely high, in excess of populations used here, suggesting that trophic niche overlap and competitive processes might be associated primarily with situations where highly abundant *P*.* parva* populations have been able to develop, such as in ponds where the bait used by anglers act as strong trophic subsidies (Jackson *et al*. 2014).

The divergence between the niches of *P*.* parva* and the native fishes was partly due to trophic position, which was generally lower in *P*.* parva*, particularly in the mesocosms and small ponds. When in sympatry with other species in mesocosms, *P*.* parva* trophic niche sizes were reduced compared with their allopatric treatment, suggesting increased diet specialization. Niche contraction often occurs with increasing interspecific competition (e.g. Svanbäck *et al*. 2008; Bolnick *et al*. 2010). These patterns were apparent with both a native species of similar size range (*G*.* aculeatus*) and in two species where body sizes tended to be larger, even when young‐of‐the‐year fish were used (*T*.* tinca*,* C*.* carpio*). Whilst we did not study wild populations of the native fishes in *P*.* parva* absence, which could be considered a deficiency of the study design, this was due to the difficulty of finding true replicates of the invaded communities but with *P*.* parva* absent. Indeed, this challenge of finding strong replicates in wild contexts was a primary reason why the mesocosm experiment was designed so that the trophic interactions of the species could be understood under simplified, replicated and controlled conditions.

The outputs of the trophic interactions between *P*.* parva* and the native fishes are contrary to the paradigm of fish invasion ecology that suggests adverse impacts often develop through increased interspecific competition for food resources between invasive and sympatric native fishes (Gozlan *et al*. 2010a; Cucherousset *et al*. 2012). Given that *P*.* parva* populations tend to be dominated by a large proportion of individuals below 50 mm that inhabit the littoral zone (Britton *et al*. 2007; Britton, Davies & Harrod 2010), intuitively, this suggests considerable potential for resource sharing with other littoral fishes. If these resources are limiting, then where the species belong to the same ecological guild, they can only coexist if there are differences in their responses to these resources (Schulze *et al*. 2012). For example, in piscivorous fishes, specialized species may persist only if their competitors are generalists in the feeding specialization continuum (Schulze *et al*. 2012). However, in our mesocosm study, the reductions in *P. parva* trophic niche size in all sympatric treatments compared to allopatry suggested that the invaders became more specialized in their diets at the population level, with this enabling their coexistence with native fish in small systems where resources could otherwise have been limiting (Chesson 2000; Kylafis & Loreau 2011). Similarly, Bolnick *et al*. (2010) found that the niche width of *G*.* aculeatus* was smaller when they coexisted with juvenile cut‐throat trout (*Oncorhynchus clarki*) and attributed the difference to an increase in among‐individual variation as a result of release from competition. The strong patterns of niche differentiation encountered here with *P*.* parva* in the mesocosms suggest that their dietary specialization was enabled through their functional traits being sufficiently plastic to enable them to differentiate in either resource use (Jackson & Britton 2014) or foraging habitat from the other fishes (Werner & Hall 1977; Robinson *et al*. 1993; Borcherding *et al*. 2013; Negus & Hoffman 2013).

An issue with many experimental approaches in ecology is that patterns measured under controlled conditions in relatively short timeframes might not necessarily match those that would develop in larger systems over longer timeframes due to issues relating to the scaling up of experimental data to represent more complex natural situations (Korsu, Huusko & Muotka 2009; Spivak, Vanni & Mette 2011). Mesocosm experiments are frequently used to understand large‐scale ecological processes, such as how nutrient enrichment affects algal communities (Spivak, Vanni & Mette 2011). Studies have shown that the outputs of these studies are often highly consistent and relevant for understanding large‐scale processes, but with the benefit of more controlled conditions and greater replication (Spivak, Vanni & Mette 2011). Our mesocosm outputs were also consistent with larger‐scale systems, with patterns detected over short time periods in mesocosms generally reflecting some of the larger‐scale patterns observed in more complex fish communities involving other non‐native fishes. This is important, as the greater replication and control provided in the mesocosms were advantageous in showing the increased dietary specialization in *P. parva* in sympatric treatments. Had treatments also included the native fishes together in sympatric contexts, similar patterns to those detected in *P*.* parva* might also have been detected, but this was not possible due to logistical constraints through the high number of mesocosms this would have required. However, it does suggest that had a functionally similar native cyprinid fish being introduced rather than *P*.* parva*, the outputs could have been similar; that is, the issue is broader than invasion ecology alone, and includes fish stocking *per se* as well as the testing of ecological theory more generally.

An aspect of the mesocosm experiment that was not tested was the degree to which resource competition in the fishes was structuring the food web. Whilst prey community structure would have been an important aspect underpinning the trophic relationships of the fishes, the densities of fish being used were designed to be sufficiently low as to not result in prey community depletion. The use of eight individuals as the number of starting fishes in each mesocosm was related to the use of eight fish in the low‐density treatments of Jackson, Ruiz‐Navarro & Britton (2015), who tested the effect of *P*.* parva* population densities on a range of their prey species. Their outputs revealed that the impact of eight fishes on their prey communities in the mesocosms was insignificant and, in the present study, was therefore unlikely to have caused complete resource depletion.

In summary, the use of *P*.* parva* as a model invasive species revealed that predicting ecological impacts that occur in wild systems following invasion can be reliably informed from small‐scale experiments in mesocosms that are reduced in complexity but increased in control and replication. We demonstrated that the result of introducing *P*.* parva* into mesocosms containing a native fish species was niche divergence with this also apparent in the invaded wild fish communities. Thus, rather than acting as a strong competitor, invasive *P*.* parva* consistently showed patterns of niche divergence that ultimately facilitated both their establishment and coexistence with other species across three distinct spatial scales.

## Data accessibility

Data available from the Dryad Digital Repository: http://dx.doi.org/10.5061/dryad.12344 (Tran *et al*. 2015).
